# Cognitive Rehabilitation as a Possible Therapeutic Approach in Patients with Blepharospasm

**DOI:** 10.3390/jcm14082574

**Published:** 2025-04-09

**Authors:** Francesco Marchet, Daniele Belvisi, Giorgio Leodori, Flavia Aiello, Matteo Costanzo, Federica Satriano, Antonella Di Vita, Antonella Conte, Giovanni Fabbrini, Gina Ferrazzano

**Affiliations:** 1 Department of Human Neuroscience, Sapienza University of Rome, Viale dell’Università 30, 00185 Rome, RM, Italy; francesco.marchet@uniroma1.it (F.M.); daniele.belvisi@uniroma1.it (D.B.); giorgio.leodori@uniroma1.it (G.L.); flavia.aiello@uniroma1.it (F.A.); federica.satriano@uniroma1.it (F.S.); antonella.divita83@gmail.com (A.D.V.); antonella.conte@uniroma1.it (A.C.); gina.ferrazzano@uniroma1.it (G.F.); 2 IRCCS Neuromed, via Atinense 18, 86077 Pozzilli, IS, Italy; 3 Department of Neuroscience, Istituto Superiore di Sanità, Viale Regina Elena 299, 00161 Rome, RM, Italy; matteo.costanzo@uniroma1.it

**Keywords:** blepharospasm, cognitive training, writing, reading, dystonia, blink rate

## Abstract

**Background/Objectives:** Blepharospasm (BSP) is a focal dystonia characterized by involuntary, bilateral spasms of the orbicularis oculi muscle. While botulinum toxin (BoNT) is the standard treatment, cognitive tasks such as reading and writing may exert transient modulatory effects on spontaneous blinking and dystonic spasms. This study investigates the potential of cognitive training, including reading and writing tasks, as a complementary therapeutic approach to BoNT in BSP patients. **Methods:** A total of 16 BSP patients were randomly assigned to two groups: Group A (n = 6) received cognitive training alongside BoNT, while Group B (n = 10) received only BoNT. Cognitive training included structured reading and writing exercises over three months. Blink rate (BR) and dystonic spasms were assessed at baseline (T0), one month (T1), and three months (T2) post-treatment. **Results:** Both groups exhibited a significant reduction in BR at T1 (*p* = 0.001), but Group A exhibited a greater improvement in BR (45.4%) compared to that of Group B (12.6%, *p* = 0.04). Reading and writing tasks were the most effective in reducing BR and dystonic spasms (*p* < 0.001). No significant correlation was found between the clinical and demographic features (*p* > 0.05). Conclusions: Cognitive training significantly enhances the therapeutic effects of BoNT on BR in BSP patients, suggesting its potential as a non-invasive complementary intervention. These preliminary findings warrant further investigation using larger cohorts and employing neurophysiological assessments.

## 1. Introduction

Blepharospasm (BSP) is a focal dystonia characterized by involuntary, bilateral, and symmetrical spasms of the orbicularis oculi (OO) muscle [1,2]. BSP presents with clinical heterogeneity. Dystonic spasms have been classified as short when lasting less than 3 s and prolonged when exceeding 3 s [1,3]. In addition to OO muscle spasms, patients with BSP may also exhibit increased blinking [4]. A five-year follow-up study conducted by our group showed that increased blinking, in the absence of OO muscle spasms, could be considered a prodromal phase of BSP [5].

From a pathophysiological perspective, studies using blink reflex recovery cycles have shown that BSP patients exhibit hyperexcitability of the brainstem circuits, likely due to reduced inhibitory control of the descending dopaminergic pathways [5,6,7,8]. Further evidence suggests that altered excitability in the brainstem circuits appears only when dystonic spasms arise and is absent in patients who exhibit increased blinking without dystonic spasms [5]. Moreover, different degrees of brainstem excitability are associated with different types of OO muscle spasms (i.e., short or prolonged spasms) [3].

Since increased blinking is part of the phenotypic spectrum of BSP, several studies have utilized blink rate (BR) as a parameter to assess the response to botulinum toxin (BoNT) treatment [9,10] or as a predictive factor for evaluating clinical outcomes following BoNT administration [11]. Furthermore, some authors have used BR, measured via surface electromyography, to assess the potential therapeutic effects of FL-41-tinted lenses in BSP patients [12]. Their findings indicated that FL-41 lenses significantly reduced mean BR compared to both rose-tinted and gray-tinted lenses and decreased eyelid contraction force compared to that observed for rose-tinted lenses.

Several studies have shown that spontaneous blinking is influenced by external factors such as natural and artificial light, the composition of the tear film, and verbal activities, including reading and speaking [4]. In line with these findings, our group has demonstrated that verbal tasks, such as reading and writing, reduce both the number of blinks and dystonic spasms. This modulation may be attributed to the activation of occipital areas during these visuo–verbal tasks, which, in turn, exert inhibitory control over the brainstem circuits responsible for dystonic spasms [9].

Based on these findings, we hypothesized that verbal tasks, such as reading and writing, could serve as a potential rehabilitative strategy for BSP patients, complementing BoNT treatment, which remains the first-line therapy for dystonia [13,14]. Thus, the aim of this study was to evaluate, through a specific rehabilitation training program, the short- and long-term effects of reading and writing on the number of OO muscle spasms and spontaneous blinks in BSP patients undergoing BoNT treatment.

## 2. Materials and Methods

### 2.1. Participants

Sixteen consecutive patients with idiopathic BSP were enrolled at the Movement Disorders Center, Department of Human Neurosciences, Sapienza University of Rome. We included BSP patients with mild disease severity (i.e., those with increased blinking and short OO muscle spasms, without prolonged spasms), as patients with prolonged OO muscle spasms represent an advanced stage of BSP in which training approaches are unlikely to be effective due to established and consolidated alterations in brainstem excitability, which is difficult to modulate. We excluded individuals with a history of exposure to dopamine receptor-blocking agents in the six months prior to enrollment, as well as those with previous or current ophthalmological disorders, including inflammatory eye diseases (e.g., blepharitis, conjunctivitis, keratitis), glaucoma, recent cataract surgery, keratoconus, or contact lens use. Patients with significant cognitive impairment (MOCA score < 26) were also excluded. Additionally, BSP patients with neurological abnormalities other than Meige syndrome or tremor—both considered part of the dystonic phenotype—were excluded. All participants were enrolled at least four months after their last BoNT treatment. Written informed consent was obtained from all patients after a thorough explanation of the study procedures. The study was designed and conducted in accordance with the ethical principles of the Declaration of Helsinki and received approval from the local ethics committee.

### 2.2. Clinical Evaluation

The Blepharospasm Severity Rating Scale (BSRS) was used to assess disease severity and the predominant clinical phenotype (increased blinking and short or prolonged dystonic spasms) [1]. Participants were videotaped during clinical evaluations, according to a standardized procedure [1]. Video recordings were conducted in a quiet room with artificial light. The camera was positioned on a tripod at a fixed distance of 2 m, focusing on the eyes. Spontaneous blinking was quantified as the BR, expressed as the number of blinks per minute. Two independent movement disorder specialists (D.B., G.F.), blinded to group assignment, reviewed the video recordings and measured the number of dystonic spasms and spontaneous blinks.

### 2.3. Experimental Protocol

In this single-blind study, patients were randomly assigned to two subgroups. Group A (6 patients) underwent cognitive training in addition to BoNT treatment, while Group B (10 patients) received only BoNT treatment. Video recordings were captured at baseline (T0) and one month after BoNT injection (T1). Patients in Group A underwent further assessment at the end of the rehabilitation program (T2, three months after T0).

### 2.4. Cognitive Training

For Group A, cognitive training lasted three months. Participants performed the activities independently at home, four times per week, and attended one supervised session per week at the Department of Human Neurosciences. These supervised sessions were led by an experienced neurologist and neuropsychologist, who also monitored the activities performed at home. To determine whether changes in dystonic spasms and BR were specifically due to reading and writing tasks rather than general occipital area activation, BSP patients also completed a control experiment involving a non-verbal visual attention training task.

Each training session, lasting approximately 30 min, included the following tasks:

1. Reading a text: Participants read aloud selected texts appropriate to their educational level. To monitor home performance, participants recorded their reading sessions (duration: 15 min).
2. Text dictation: Participants listened to audio recordings of selected texts and transcribed them (duration: 10 min).
3. Text-error detection task: Participants were presented with texts containing spelling errors and were asked to identify and underline them (duration: 5 min).

During the weekly session at our center, the above tasks were supplemented with two additional tasks (each lasting approximately 15 min):

- A visual attention task, in which participants observed a dot moving horizontally across a screen and pressed a button when the distance between two consecutive positions exceeded the standard distance.
- An auditory attention task, in which participants listened to a randomized list of common words and pressed a button when they heard a previously assigned target word.

### 2.5. Statistical Analysis

All statistical analyses were performed using the Statistical Package for the Social Sciences (SPSS 25.0, IBM, New York, NY, USA). Descriptive statistics were calculated for demographic and clinical characteristics. The Shapiro–Wilk test was used to assess the normality of continuous variables, reported as means ± standard deviations. Between-group comparisons were conducted using a Student’s *t*-test for continuous variables with a normal distribution (age, disease duration, BoNT duration, BSRS) and the Chi-square test for categorical variables (sex). A paired t-test was used to compare measurements before and after one month and before and after three months for each individual, while an independent *t*-test was used to compare the two groups (Group A vs. Group B). The percentage of improvement was calculated using the following formula:

((baseline − T1)/baseline) × 100

One-way ANOVA was used to compare the effect of each cognitive task on the number of blinks and the number of dystonic spasms. Correlations between demographic features and clinical characteristics were assessed using Spearman’s correlations. *p*-values < 0.05 were considered statistically significant.

## 3. Results

The demographic and clinical data of BSP patients are summarized in Table 1. At baseline, no significant differences were observed between the two groups (Group A vs. Group B) in terms of demographic characteristics, disease severity, BR, or the number of dystonic spasms (all *p* > 0.05).

Each enrolled BSP patient (in both Group A and Group B) showed a significant improvement in BR at the one-month follow-up (*p* = 0.001), while no significant differences were found in the number of dystonic spasms (*p* = 0.33). The BSRS score showed a trend towards significance (*p* = 0.055) in all BSP patients enrolled (both in Group A and B) after one month of treatment. At the one-month follow-up, the percentage of improvement in BR was significantly greater in Group A (45.4%) compared to in Group B (12.6%, *p* = 0.04). Data are shown in Table 2.

**Figure 1:**
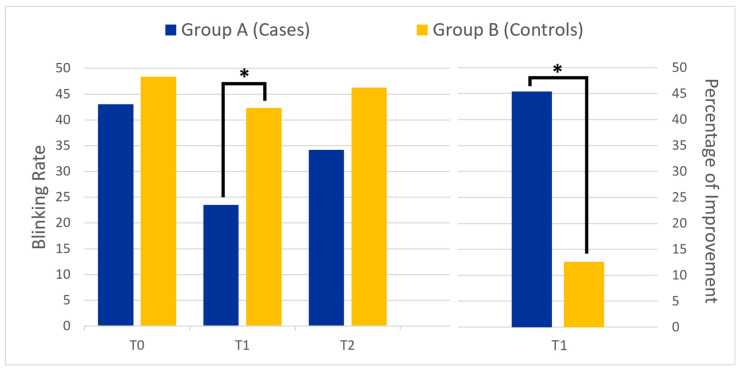
The left panel shows the mean blink rate at different time points for the two groups: before botulinum toxin injection (T0), one month after (T1), and three months after treatment (T2). The right panel compares the percentage of improvement at T1 between the two groups. The asterisk (*) indicates statistical significance.

The two groups differed only in regards to BR at one month after treatment (*p* = 0.03, see Figure 1). When comparing the cognitive tasks used during training, we found that reading and writing were the two tasks that led to a significant reduction in both the number of blinks and dystonic spasms (*p* < 0.001).

At the three-month follow-up evaluation (T2), we found that BR and the number of dystonic spasms in Group A and in Group B remained unchanged compared to the baseline values (*p* > 0.05).

No significant correlations were found between clinical features (BR, dystonic spasms, or disease severity) and demographic characteristics (all *p* > 0.05) in either group.

## 4. Discussion

This pilot study demonstrated that the combination of cognitive training and BoNT treatment resulted in a greater reduction in BR compared to that for BoNT treatment alone. Among the tasks used during cognitive training, reading and writing produced the most notable improvements in blink rate. While the combined therapeutic approach significantly reduced BR, it showed only a trend toward significance in reducing the number of dystonic spasms and disease severity at the one-month follow-up. At the three-month follow-up, both BR and the number of dystonic spasms remained stable in Groups A and B.

We took several precautions to minimize bias that could influence our results. Video recordings were conducted in a controlled, comfortable environment with constant light intensity, and participants with ophthalmological conditions affecting dystonic spasms or BR were excluded. Considering the influence of dopamine levels on BR, we also excluded individuals taking dopaminergic medications. Finally, all patients were assessed at least four months following their last BoNT injection to eliminate any residual treatment effects.

To our knowledge, this is the first study to demonstrate that prolonged cognitive training with reading and writing tasks induces a long-lasting modulation of BR in BSP patients—an effect that significantly surpasses that of BoNT treatment alone. Based on previous evidence, the neurophysiological substrate of BSP originates from hyperactive brainstem neural generators, which are regulated by cortical and subcortical structures [6]. Specifically, increased excitability of the trigeminal–facial brainstem circuit—potentially due to diminished basal ganglia inhibition—leads to dystonic spasms. This circuit receives excitatory inputs from the substantia nigra and superior colliculus and inhibitory inputs from the cerebellum and occipital cortex [15,16]. Although the precise neural substrates of spontaneous blinking remain partially unclear, evidence suggests a key role for the spinal trigeminal complex, modulated by the paramedian pontine reticular formation, as well as contributions from the mesial frontal region [17,18].

Therefore, we hypothesize that our results might be due to the inhibitory effects exerted by verbal tasks on the occipital cortex, a key region involved in visual attention and fixation, which has also been implicated in blink reflex modulation [19]. A possible pathophysiological mechanism for this effect might involve the lingual gyrus, a key occipital structure associated with visual processing, which has been implicated in the modulation of BSP severity. A study by Glickman et al. [20] identified co-activation of the lingual gyrus with spasm severity, indicating that this region may be critically involved in the persistence or exacerbation of dystonic symptoms. Therefore, we might speculate that given that the lingual gyrus plays a fundamental role in visuomotor integration and fixation stability, its activation during cognitive tasks requiring sustained visual engagement—such as reading and writing—could contribute to the observed reduction in BR. This suggests that inhibitory projections from the occipital cortex could modulate trigeminal–facial brainstem circuits, either through direct suppression of facial motoneurons or via indirect pathways involving the basal ganglia. In this context, the basal ganglia, particularly the substantia nigra pars reticulata, may serve as a critical relay for inhibitory control. The substantia nigra has been shown to influence both the superior colliculus, which processes visual information, and the nucleus raphe magnus, which projects to the trigeminal blink reflex circuits [21]. Dysfunction within this network has been implicated in the pathophysiology of BSP, further supporting the hypothesis that cognitive training may modulate existing neural circuits to produce a therapeutic effect.

Another finding of our study was the lack of improvement in the number of dystonic spasms or in the BSRS total score one month after treatment. This could be because the scale items primarily evaluate the severity of spasms, especially prolonged ones, which have a greater impact on the total score, and our patients predominantly exhibited increased blinking and brief spasms. This is supported by the observation that even in the group of patients treated with only BoNT, the same result was obtained.

Finally, three months after enrollment (T2), we did not observe any changes in BR or in the number of dystonic spasms in Group A. These results may be due to the small sample size (only six patients). Future studies are needed to investigate the potential long-lasting effects of cognitive training beyond those combined with the impact of BoNT.

Our study has several limitations. First is the small sample size. However, this is a pilot study on BSP, which is a rare disease, and we recruited only patients with mild forms of BSP (increased blinking or brief spasms). Future studies using larger samples will be needed to confirm our data. Moreover, we did not include neurophysiological correlations to assess the effects of cognitive training on the structures modulating the blink and spasm generators. Future investigations should evaluate whether these modulatory changes are associated with improvements in neurophysiological measures such as the blink reflex recovery cycle. Another limitation of this study is the unequal group sizes (Group A: 6 patients; Group B: 10 patients). Given the relatively small sample size and the exploratory nature of this pilot study, we opted for a simple randomization method without stratification. Moreover, the inclusion criteria were stringent, as we specifically selected patients with mild forms of BSP characterized predominantly by increased blinking and short OO muscle spasms. This selection process, combined with randomization, led to an unequal final distribution. However, despite the difference in sample sizes, no significant baseline differences were observed between the two groups in terms of demographic characteristics, disease severity, BR, or the number of dystonic spasms. This indicates that the randomization process did not introduce any systematic bias. Future studies with larger cohorts and a stratified randomization approach will be necessary to confirm and expand upon our findings while ensuring more balanced group sizes.

In conclusion, our study demonstrated that prolonged verbal cognitive training, when used as an add-on to BoNT treatment, produces a significant and long-lasting reduction in the number of blinks in BSP patients. These findings provide novel insights into the potential of non-invasive interventions for BSP, expanding the current understanding of its neurophysiological basis, and suggest that cognitive interventions may represent a promising complementary therapeutic strategy.

## Figures and Tables

**Table 1:** Demographic and clinical characteristics of BSP patients.

**Group A**	**Sex**	**Age** **(y)**	**Disease** **Duration** **(y)**	**BoNT** **Duration** **(y)**	**BSRS** **T0**	**BSRS** **T2**
**1**	M	75	8	4	5	6
**2**	M	69	10	5	4	10
**3**	M	60	2	1	4	2
**4**	F	73	5	3	2	3
**5**	F	63	12	8	10	4
**6**	F	70	10	6	6	3
**Mean ± SD**		68 ± 5.8	7.8 ± 3.7	4.5 ± 2.4	5.2 ± 2.7	4.6 ± 2.9
**Group B**	**Sex**	**Age** **(y)**	**Disease** **Duration** **(y)**	**BoNT** **Duration** **(y)**	**BSRS** **T0**	**BSRS** **T2**
**7**	F	62	7	4	4	4
**8**	F	87	14	11	5	5
**9**	M	61	8	2	13	10
**10**	F	79	11	7	3	2
**11**	M	75	23	20	4	4
**12**	M	70	4	2	4	4
**13**	F	64	5	2	3	3
**14**	M	63	3	2	4	3
**15**	M	64	10	4	6	6
**16**	F	66	6	3	4	4
**Mean ± SD**		69.1 ± 8.62	9.1 ± 5.9	5.7 ± 5.8	5.0 ± 3.0	4.5 ± 2.2

The table presents individual data alongside the group mean ± standard deviation. Between-group comparisons were performed using a Student’s *t*-test for normally distributed continuous variables (age, disease duration, BoNT duration, BSRS), and a Chi-square test for categorical variables (sex). F: female; M: male; BoNT: botulinum toxin; Y: years; BSRS: Blepharospasm Severity Rating Scale; T0: baseline; T2: three months after treatment.

**Table 2:** Comparison between the two groups in terms of blink rate, dystonic spasms, and Blepharospasm Severity Rating Scale.

	Group A	Group B	*p* Value
**T0**			
**BR**	43.06 ± 19.54	48.40 ± 22.56	0.52
**Dystonic Spasms (n)**	12.45 ± 14.97	27.80 ± 25.85	0.16
**BRSR**	5.00 ± 2.76	5.00 ± 2.94	0.82
**T1**			
**BR**	23.50 ± 12.99	42.30 ± 18.56	0.001
**Dystonic Spasms (n)**	7.85 ± 9.16	17.30 ± 20.68	0.33
**BRSR**	4.83 ± 2.86	4.60 ± 2.50	0.055
**T2**			
**BR**	34.17 ± 17.97	46.30 ± 24.01	0.57
**Dystonic Spasms (n)**	9.33 ± 11.38	16.20 ± 16.96	0.55
**BRSR**	4.67 ± 2.94	4.50 ± 2.22	0.39
**% change T0–T1**			
**BR**	45.42%	12.60%	0.04
**Dystonic Spasms (n)**	36.94%	37.79%	0.82
**BRSR**	3.40%	8.00%	0.62
**% change T0–T2**			
**BR**	20.64%	4.30%	0.052
**Dystonic Spasms (n)**	25.06%	41.72%	0.06
**BRSR**	6.60%	10.00%	0.07

BR: blink rate; n: number; BSRS: Blepharospasm Severity Rating Scale; T0: baseline; T1: one month after treatment; T2: three months after treatment.

## Data Availability

Data are available upon reasonable request to the corresponding author.

## Abbreviations

The following abbreviations are used in this manuscript:

BSP	blepharospasm
OO	orbicularis oculi
BoNT	botulinum neurotoxin
BR	blink rate

## References

[1] Defazio G., Hallett M., Jinnah H.A., Berardelli A. (2013). Development and Validation of a Clinical Guideline for Diagnosing Blepharospasm. Neurology.

[2] Albanese A., Bhatia K., Bressman S.B., Delong M.R., Fahn S., Fung V.S.C., Hallett M., Jankovic J., Jinnah H.A., Klein C. (2013). Phenomenology and Classification of Dystonia: A Consensus Update. Mov. Disord. Off. J. Mov. Disord. Soc..

[3] Defazio G., Hallett M., Jinnah H.A., Stebbins G.T., Gigante A.F., Ferrazzano G., Conte A., Fabbrini G., Berardelli A. (2015). Development and Validation of a Clinical Scale for Rating the Severity of Blepharospasm. Mov. Disord. Off. J. Mov. Disord. Soc..

[4] Bentivoglio A.R., Daniele A., Albanese A., Tonali P.A., Fasano A. (2006). Analysis of Blink Rate in Patients with Blepharospasm. Mov. Disord. Off. J. Mov. Disord. Soc..

[5] Conte A., Ferrazzano G., Defazio G., Fabbrini G., Hallett M., Berardelli A. (2017). INCREASED BLINKING MAY BE A PRECURSOR OF BLEPHAROSPASM: A LONGITUDINAL STUDY. Mov. Disord. Clin. Pract..

[6] Berardelli A., Rothwell J.C., Day B.L., Marsden C.D. (1985). Pathophysiology of Blepharospasm and Oromandibular Dystonia. Brain J. Neurol..

[7] Jinnah H.A., Berardelli A., Comella C., Defazio G., Delong M.R., Factor S., Galpern W.R., Hallett M., Ludlow C.L., Perlmutter J.S. (2013). The Focal Dystonias: Current Views and Challenges for Future Research. Mov. Disord. Off. J. Mov. Disord. Soc..

[8] Tolosa E., Montserrat L., Bayes A. (1988). Blink Reflex Studies in Focal Dystonias: Enhanced Excitability of Brainstem Interneurons in Cranial Dystonia and Spasmodic Torticollis. Mov. Disord. Off. J. Mov. Disord. Soc..

[9] Ferrazzano G., Conte A., Belvisi D., Fabbrini A., Baione V., Berardelli A., Fabbrini G. (2019). Writing, Reading, and Speaking in Blepharospasm. J. Neurol..

[10] Yazdanpanah G., Yen M.T., Pflugfelder S.C. (2023). Quantitative assessment of botulinum toxin injection on blink rate in blepharospasm. Orbit.

[11] Jang J., Lew H. (2021). Blink index as a response predictor of blepharospasm to botulinum neurotoxin-A treatment. Brain Behav..

[12] Blackburn M.K., Lamb R.D., Digre K.B., Smith A.G., Warner J.E., McClane R.W., Nandedkar S.D., Langeberg W.J., Holubkov R., Katz B.J. (2009). FL-41 tint improves blink frequency, light sensitivity, and functional limitations in patients with benign essential blepharospasm. Ophthalmology.

[13] Simpson D.M., Hallett M., Ashman E.J., Comella C.L., Green M.W., Gronseth G.S., Armstrong M.J., Gloss D., Potrebic S., Jankovic J. (2016). Practice Guideline Update Summary: Botulinum Neurotoxin for the Treatment of Blepharospasm, Cervical Dystonia, Adult Spasticity, and Headache: Report of the Guideline Development Subcommittee of the American Academy of Neurology. Neurology.

[14] Albanese A., Colosimo C., Carretta D., Dickmann A., Bentivoglio A.R., Tonali P. (1992). Botulinum Toxin as a Treatment for Blepharospasm, Spasmodic Torticollis and Hemifacial Spasm. Eur. Neurol..

[15] Karson C.N. (1989). Blinking. Bull. Soc. Belge Ophtalmol..

[16] Chen F.-P., Evinger C. (2006). Cerebellar Modulation of Trigeminal Reflex Blinks: Interpositus Neurons. J. Neurosci. Off. J. Soc. Neurosci..

[17] Yoon H.W., Chung J.-Y., Song M.-S., Park H. (2005). Neural Correlates of Eye Blinking; Improved by Simultaneous fMRI and EOG Measurement. Neurosci. Lett..

[18] Kaminer J., Powers A.S., Horn K.G., Hui C., Evinger C. (2011). Characterizing the Spontaneous Blink Generator: An Animal Model. J. Neurosci. Off. J. Soc. Neurosci..

[19] Baker R.S., Andersen A.H., Morecraft R.J., Smith C.D. (2003). A Functional Magnetic Resonance Imaging Study in Patients with Benign Essential Blepharospasm. J. Neuro-Ophthalmol. Off. J. N. Am. Neuro-Ophthalmol. Soc..

[20] Glickman A., Nguyen P., Shelton E., Peterson D.A., Berman B.D. (2020). Basal Ganglia and Cerebellar Circuits Have Distinct Roles in Blepharospasm. Park. Relat. Disord..

[21] Peterson D.A., Sejnowski T.J. (2017). A Dynamic Circuit Hypothesis for the Pathogenesis of Blepharospasm. Front. Comput. Neurosci..

